# S100A8 and S100A9 Induce Cytokine Expression and Regulate the NLRP3 Inflammasome via ROS-Dependent Activation of NF-κB^1^

**DOI:** 10.1371/journal.pone.0072138

**Published:** 2013-08-19

**Authors:** Jean-Christophe Simard, Annabelle Cesaro, Julie Chapeton-Montes, Mélanie Tardif, Francis Antoine, Denis Girard, Philippe A. Tessier

**Affiliations:** 1 Laboratoire de Recherche en Inflammation des Granulocytes, Université du Québec Institut National de la Recherche Scientifique, Institut Armand-Frappier, Laval, Québec, Canada; 2 Axe Maladies Infectieuses et Immunitaires, Centre de Recherche du CHU de Québec and Faculté de Médecine, Université Laval, Québec, Canada; University of California, Riverside, United States of America

## Abstract

S100A8 and S100A9 are cytoplasmic proteins expressed by phagocytes. High concentrations of these proteins have been correlated with various inflammatory conditions, including autoimmune diseases such as rheumatoid arthritis and Crohn’s disease, as well as autoinflammatory diseases. In the present study, we examined the effects of S100A8 and S100A9 on the secretion of cytokines and chemokines from PBMCs. S100A8 and S100A9 induced the secretion of cytokines such as IL-6, IL-8, and IL-1β. This secretion was associated with the activation and translocation of the transcription factor NF-κB. Inhibition studies using antisense RNA and the pharmacological agent BAY-117082 confirmed the involvement of NF-κB in IL-6, IL-8, and IL-1β secretion. S100A8- and S100A9-mediated activation of NF-κB, the NLR family, pyrin domain-containing 3 (NLRP3) protein, and pro-IL-1β expression was dependent on the generation of reactive oxygen species. This effect was synergistically enhanced by ATP, a known inflammasome activator. These results suggest that S100A8 and S100A9 enhance the inflammatory response by inducing cytokine secretion of PBMCs.

## Introduction

Endogenous signals originating from stressed, injured, and necrotic cells can activate the innate and adaptive immune systems [1]. These signals have been termed as danger-associated molecular patterns (DAMPs) or alarmins [2]. Like pathogen-associated molecular patterns, DAMPs are recognized by a variety of pathogen-recognition receptors (PRRs) including the toll-like receptors, the formyl-methionine receptors, and the receptor for advanced glycation end-products (RAGE) [1]. Hyaluronan, galectins, high-mobility group box protein, IL-1, and S00A8/A9 are examples of DAMPs [2]. Most DAMPs combine intra- and extra-cellular activities. S100A8 and S100A9 are not exceptions and act as regulators of NADPH oxidase inside the cells and as pro-inflammatory factors once secreted [3], [4]. S100A8 and S100A9 are arranged as noncovalently bonded homodimers. In addition, in the presence of calcium, S100A8 and S100A9 form a noncovalent heterodimer called S100A8/A9 or calprotectin, which is presumed to be involved in the cellular control of calcium concentrations. Once released extracellularly, they participate actively in the inflammation process by promoting phagocyte migration [5]–[7]. S100A8 and S100A9 are presumed to bind to the PRRs, TLR4, and RAGE, thereby leading to transcription of pro-inflammatory genes [8], [9]. S100A9 is also a potent activator of various neutrophil functions, such as degranulation and phagocytosis [10], [11], supporting its role as a DAMP.

High extracellular concentrations of S100A8 and S100A9 are found in the serum and at inflammatory sites in autoimmune diseases including arthritis [12]–[14], lupus, and Crohn’s disease [15], [16]. In addition, recent studies have demonstrated a role for S100A9 in animal models of lupus, arthritis, and Alzheimer’s disease [17]–[20].

IL-1β is a well-known cytokine, and its effect on inflammation and in autoinflammatory disorders has been extensively studied [21]–[23]. In monocytes/macrophages, IL-1β secretion is predominantly controlled by the NLR family, pyrin domain-containing 3 (NLRP3) inflammasome (a multi-protein complex formed by the protein NLRP3), the adaptor protein called apoptotic speck protein (ASC), and the effector protein caspase-1 [24]. Upon activation of the inflammasome, pro-caspase-1 is recruited into the inflammasome complex and is auto-cleaved into its caspase-recruitment and activation domain, a p10 and a p20 fragment, respectively. Two molecules of the p10 fragment then associate with two p20 fragments to form the active heterotetramer caspase-1 enzyme that cleaves pro-IL-1β into IL-1β and allows its secretion [24].

The inflammasome is formed in cells exposed to microbial, environmental, or endogenous danger stimuli. Two signals are traditionally required for NLRP3 inflammasome activation. The first signal, for example, transmitted by lipopolysaccharide (LPS), induces pro-IL-1β transcription [25], [26]. The second required signal is provided by reduction of intracellular K^+^ [27]. Stimuli such as ATP or the microbial pore-forming toxin nigericin induce strong K^+^ efflux, thus promoting rapid activation of caspase-1 and secretion of mature IL-1β [28].

We recently reported that S100A9 induces the secretion of cytokines/chemokines in human monocytes [17]. We herein report that S100A8 and S100A9 induce the secretion of several pro-inflammatory cytokines, including IL-6, TNFα, and IL-1β, by stimulating production of reactive oxygen species (ROS). These, in turn, activate the transcription factor NF-κB, leading to cytokine secretion and expression and activation of the NLRP3 inflammasome.

## Materials and Methods

### Ethics Statement

These studies were approved by the INRS-Institut Armand-Frappier’s ethical committee, and all participants gave written informed consent.

### Reagents

Human recombinant S100A8 and S100A9 were produced as previously described [29] and found were to contain less than 1 pg endotoxin/mg of protein. LPS (from *E. coli*), cycloheximide, diphenyleneiodonium chloride (DPI), N-acetylcysteine (NAC), and anti-β-actin were purchased from Sigma-Aldrich (St. Louis, MO). BAY-117082 (BAY) was purchased from Calbiochem (San Diego, CA). Anti-phospho-inhibitory kappa B kinase (IKK)-α/β (Ser176/177), anti-phospho-IKK-γ (Ser376), and anti-caspase-1 were purchased from Cell Signaling Technology (Danvers, MA). Antibodies against IκB-α (H-4), GAPDH (FL), NF-κB p50 (4D1), NF-κB p65 (C-20), ASC and pro-IL-1β (H-153) were obtained from Santa Cruz Biotechnology (Santa Cruz, CA). The NLRP3/NALP-3 antibody was purchased from Enzo Life Sciences (Farmingdale, NY). RPMI 1640, HEPES, penicillin/streptomycin (pen/strep), heat-inactivated FBS, opti-MEM media, and HBSS were purchased from Life Technologies (Grand Island, NY). Ficoll-Paque was obtained from GE Healthcare Bio-Science AB (Uppsala, Sweden). All secondary antibodies were purchased from Jackson ImmunoResearch Laboratories (West Grove, PA). The Proteome Profiler™ Array (Human Cytokine Array Panel A) was purchased from R&D Systems (Minneapolis, MN). THP-Blue™, QuantiBlue™, and standard LPS 0111:B4 were purchased from InvivoGen (San Diego, CA). Protease Inhibitor Cocktail was purchased from Thermo Fisher Canada (Nepean, Ontario). PureProteome Protein A Magnetic Beads were obtained from Millipore (Billerica, MA).

### PBMC Isolation

Blood samples were obtained from informed, consenting individuals according to institutionally approved procedures. PBMCs were isolated from venous blood of healthy volunteers using dextran sedimentation, followed by centrifugation over a Ficoll-Paque cushion. PBMCs were harvested and washed after centrifugation. Cell viability was monitored by trypan blue exclusion and consistently found to be greater than 99%. PBMCs were resuspended at 1×10^6^ cells/ml in RPMI 1640 containing 10% autologous decomplemented serum.

### Western Blot Analyses

PBMCs were stimulated with S100A8 (10 µg/ml), S100A9 (10 µg/ml), LPS (1 µg/ml), or the equivalent volume of diluent (HBSS, 1×) at 37°C for various periods of time, as specified. For detection of IκB-α, cells were pre-treated with 5 µM cycloheximide to limit rapid turnover of the protein. At the end of the incubation period, cells were lysed in Laemmli’s sample buffer (0.25 M Tris-HCl [pH 6.8], 8% SDS, 40% glycerol, and 20% 2-ME), and aliquots of extracts (corresponding to 8×10^5^ cells) were subjected to 10% SDS-PAGE and transferred to nitrocellulose membranes for detection of specific proteins. Membranes were blocked for 1 h at room temperature in 5% milk or in BlØk-PO Reagent fromMillipore for phosphorylated proteins. After washing, primary antibodies were added at a final dilution of 1∶1000 in 0.15% TBS-Tween. The membranes were incubated overnight at 4°C, washed with TBS-Tween, and incubated for 1 h at room temperature with a goat anti-rabbit IgG HRP secondary Ab (Jackson ImmunoResearch Laboratories) diluted 1∶25,000 in TBS-Tween or a goat anti-mouse IgG HRP secondary Ab (Jackson ImmunoResearch Laboratories) diluted at 1∶25,000 in TBS-Tween. After several washes, protein expression was evaluated using the Luminata Forte Western HRP Substrate (Millipore). Membranes were stripped with ReBlot Plus Strong (Millipore) and reprobed to confirm equal loading of proteins.

### Immunoprecipitation

PBMCs (6×10^6^ cells) were stimulated for 4 hours with the indicated agonists followed by 30 min with 1 mM ATP. Cells were then centrifuged and lyzed in ice-cold lysis buffer 1X (50 mM Tris-HCl pH 8, 100 mM NaCl, 1% Triton X-100, 5 mM EDTA and Protease Inhibitor Cocktail 1X) for 1 hour on ice and sonicated 3 times for 20 s. The lysates were first incubate 1 h on ice with magnetic beads (5 µl per 10^6^ cells) for 1 h with agitation to remove non-specific binding. The beads were then removed and the samples were incubated overnight with 5 µg of rabbit anti-ASC at 4°C with agitation. Protein A magnetic beads were then added (5 µl per 1×10^6^ cells) for 30 min at R.T. with agitation. The complex (antibody, bead, protein) was collected and 50 µL of Laemmli’s sample buffer 1X was added. The samples were then boiled at 100°C for 10 min, and the immunoprecipitates were then loaded on a SDS-page gel for western blot analysis.

### EMSA

Nuclear extracts from cells stimulated for 4 h were prepared using the NucBuster protein extraction kit from EMD Millipore. The EMSA reaction was performed using the Gel Shift Assay System from Promega (Madison, WI). Briefly, 7 µg of nuclear protein was mixed with 1.75 pmol of unlabeled NF-κB consensus oligonucleotides (competitive assay) or unlabeled AP-1 consensus oligonucleotides (non-competitive assay) for 15 min at room temperature. Next, 0.03 pmol of (^32^P)-labeled NF-κB consensus oligonucleotides were added for 30 min at room temperature. The DNA-protein complex was resolved on 4% non-denaturing polyacrylamide gels. Gels were exposed for 24 h at −80°C, and the bands were visualized using X-OMAT films.

### NF-κB p50 TransFactor ELISA Assay

Assays were performed using the Colorimetric TransFactor ELISA kit (Clontech Laboratories, Mountain View, CA) according to the manufacturer’s protocol. Briefly, nuclear extracts were prepared as described above. Nuclear proteins (25 µg) were incubated with or without competitor oligonucleotides for 15 min on ice. Competitor oligonucleotides correspond to the same DNA sequence as that of the oligo-coated wells, showing the binding specificity between DNA and the transcription factor. Samples were then added to wells for 60 min at room temperature. After four washes, primary antibody was added to each well and incubated for 60 min at room temperature. After four washes, a goat-anti-rabbit IgG-HRP secondary antibody was added to each well and incubated for 30 min at room temperature. After four washes, the tetramethylbenzidine (TMB) substrate was added to each well for 10 min at room temperature. Finally, 1 M H_2_SO_4_ was added to each well, and absorbance was read at 450 nm using a SpectraMax M5 spectrophotometer (Molecular Devices, Sunnyvale, CA.

### Antisense Experiments

PBMCs (1×10^6^/ml) were incubated with p50 (5′-tggatcttctgccattct-3′), p65 (5′-ggggaacagttcgtccatggc-3′), or scrambled (5′-ttaccgcgccgtagacgggca-3′) antisense oligonucleotides (AS) with a phosphorothioate backbone at a final concentration of 10 µM or 40 µM at 37°C in serum-free Opti-MEM media (Invitrogen) for 4 h to increase oligonucleotide uptake. Subsequently, cells were stimulated with agonists for 20 h in Opti-MEM media supplemented with 5% FBS. The transfection efficiency was greater than 90%, as determined using the same oligonucleotide sequences with a Cy3 fluorophore coupled to the 5′ end and visualized by fluorescence microscopy. p50 and p65 protein expression was monitored by western blot analysis.

### Immunofluorescence Microscopy

As described previously [30], following the incubation of Cy5-AS, cells were washed twice in ice-cold PBS and cytocentrifuged on glass coverslips (Fisher Scientific, Ottawa, Canada). Coverslips were mounted using ProLong Gold Antifade Media containing DAPI (Invitrogen). Fluorescent-labeled cells were captured from high-power field (3400) and observed using a Leica microscope equipped with an ebq 100 dc epifluorescent condenser.

### Proteome Profiler™ Array

The Human Cytokine Array Panel A (Proteome Profiler™ Array) was purchased from R&D Systems. All steps were performed within 3 weeks of harvesting the cells. Cells from 10 different PBMC donors were stimulated for 24 h with the corresponding agonists. Supernatants were pooled together and used to probe the membranes. The chemiluminescent signals from the bound cytokines present in the supernatants were detected on X-OMAT films. Values obtained from densitometry analyses were considered when above 9000 units, which corresponds to the background value.

### Caspase-1 Assay

Caspase-1 activity was measured using the Caspase-1 Colorimetric Assay kit (R&D Systems) according to the recommended protocol. Briefly, 2×10^6^ cells were stimulated with agonists for the indicated time period. Cells were lysed in ice-cold lysis buffer and incubated on ice for 10 min. Cells were centrifuged at 10,000 g for 1 min. Supernatants were then used for enzymatic reactions. Caspase-1 colorimetric substrate (WEHD-pNA) from the kit was added to each reaction mix with the corresponding volume of cell lysate, reaction buffer, and DTT. Plates were incubated for 2 h at 37°C. After incubation, plates were read on a microplate reader at a wavelength of 405 nm. Results are expressed as fold-increase in caspase-1 activity.

### IL-6, IL-8, and IL-1β Production

IL-6, IL-8, and IL-1β levels were measured using commercially available ELISA kits (Invitrogen). Freshly isolated cells were incubated with S100A8 (10 µg/ml), S100A9 (10 µg/ml), LPS (1 µg/ml), or the diluent (HBSS) for the indicated time period in 96-well plates containing RPMI-1640 supplemented with 10% autologous serum. Supernatants were harvested after centrifugation and stored at –80°C before ELISA.

### Measurement of NF-κB Activity Using THP1-XBlue™ Cells

THP1-XBlue™ cells (1×10^5^) were pretreated with DPI (10 µM) or vehicle (DMSO) for 1 h and then stimulated with S100A8 (10 µg/ml), S100A9 (10 µg/ml), or LPS (1 µg/ml) for 24 h. Supernatants were harvested and incubated with Quanti-Blue™, which turns purple in the presence of secreted alkaline phosphatase (SEAP). SEAP levels were determined spectrophotometrically at 650 nm.

### siRNA Transfection

PBMCs were transfected with Silencer Select Pre-designed siRNA from Ambion (Austin, TX) according to the manufacturer’s protocol. Briefly, cells were suspended in 500 µL of opti-MEM reduced serum medium containing 3 µL of Lipofectamine RNAiMAX Reagent (Life Technologies) and various concentration of siRNA. SiRNA targeting ASC (sense UGAUCUUUUUAUACACAAUTT, antisense AUUGUGUAUAAAAAGAUCAGA), NLRP-3 (sense GGAGAGACCUUUAUGAGAATT, antisens UUCUCAUAAGGUCUCUCCTG) or Control non-silencingsiRNA (sense UUCUCCGAACGUGUCACGUTT, antisense ACGUGACACGUUCGGAGGAGAATT) were used.After 24 hours, cells were washed and stimulated with the indicated agonists.

### Statistical Analyses

Experimental data are expressed as means ± SEM. One-way ANOVA (Dunnett’s multiple-comparison test) and two-way ANOVA (Bonferroni’s post-test) were performed using Graph-Pad Prism (version 5.01). Differences were considered statistically significant as follows: * p≤0.05, **p≤0.01, and ***p≤0.005 versus buffer or the appropriate diluent. Densitometric analyses were performed using AlphaEaseFC (FluorChem HD2).

## Results

### S100A8 and S100A9 Induce Inflammatory Cytokine Secretion

**Figure 1:**
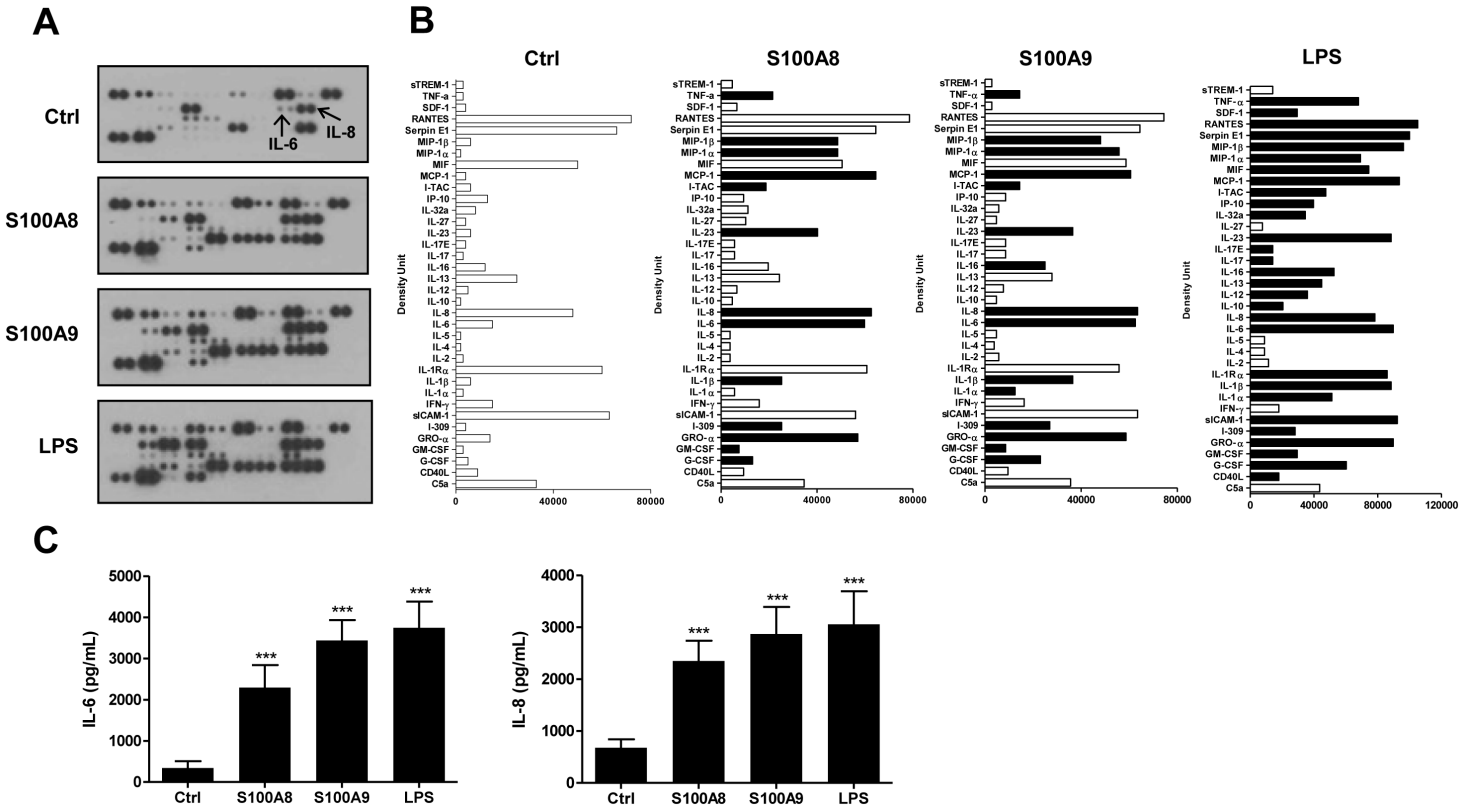
S100A8 and S100A9 induce the secretion of several cytokines in human PBMCs. Cytokine production was evaluated using proteome profiler arrays (A and B) or ELISA (C), as described in Materials and Methods. Cells were stimulated for 24 hours, and supernatants were harvested and used for experiments. (B) Densitometry analyses of antibody arrays in PBMCs. Data represent supernatants pooled from 10 different donors (A and B). Black bars, upregulated cytokines; white bars, unchanged cytokines. Data are the mean ± SEM of at least five experiments performed on cells from different donors (C). ***p≤0.005, Dunnett’s multiple comparison test.

The effect of S100A8 and S100A9 on the production of cytokines/chemokines was first screened using an antibody array. As illustrated in Figure 1A and 1B, S100A8 and S100A9 induced the secretion of several pro-inflammatory cytokines in PBMCs (**Fig. 1A and 1B**). Among these, the cytokines IL-6, IL-1β, TNF-α and the chemokines IL-8, growth-regulated oncogene alpha (Gro-α), MIP-1α, and MIP-1β were the most secreted. The secretion of IL-6 and IL-8 in PBMCs was also confirmed by ELISA (**Fig. 1C**). The concentrations of these cytokines were comparable to those observed in the supernatants of cells stimulated with LPS. Isolation of monocytes, CD4+ T cells, CD8+ T cells, and B cells revealed that monocytes were the only cell type responsible for cytokine production (data not shown).

### S100A8 and S100A9 Increase NF-κB Translocation and Activation

**Figure 2:**
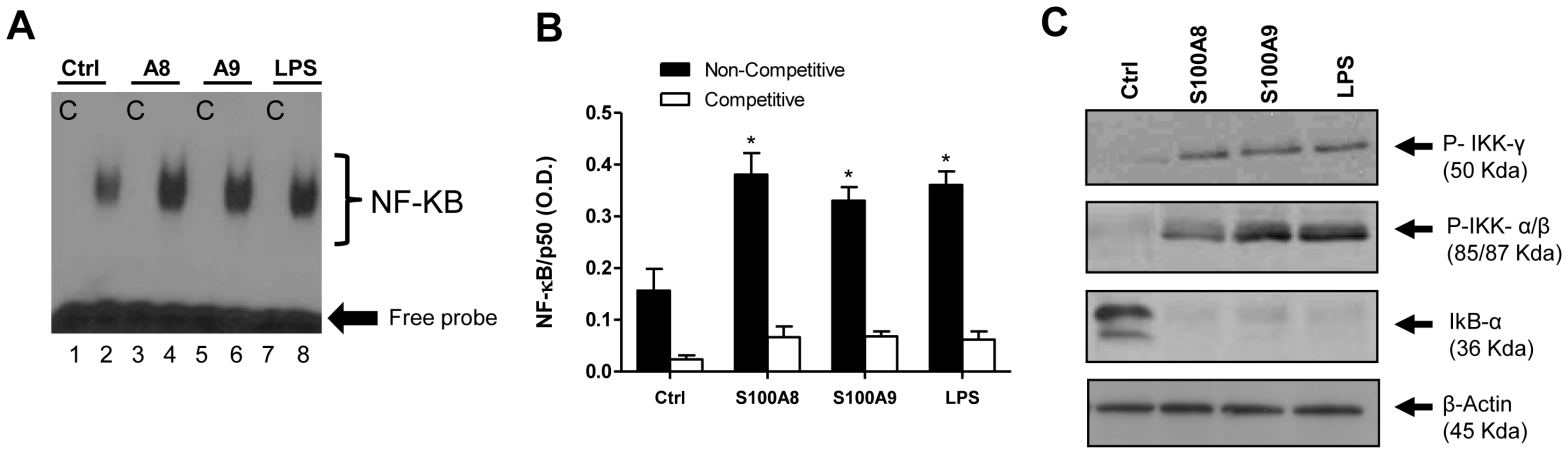
S100A8 and S100A9 increase translocation of NF-κB in human PBMCs. NF-κB translocation and binding were assessed by EMSA (A) and by a NF-κB p50 TransFactor ELISA kit (B). Nuclear proteins were isolated from cells stimulated for 4 h. Lanes 1, 3, 5, and 7 (c, competitive assay). Lanes 2, 4, 6, and 8 (non-competitive assay). Phosphorylation of the IKK complex and degradation of IκB-α were assessed by immunoblotting (C). Results shown are of one experiment that is representative of at least four other experiments (A and C). Data are the mean ± SEM of three experiments performed on cells from different donors (B). *p≤0.05, Dunnett’s multiple comparison test.

The production of pro-inflammatory cytokines and chemokines largely depends on transcription factors such as NF-κB and AP-1. Activation of NF-κB in S100A8- and S100A9-treated PBMCs was therefore examined using EMSA. As shown in Figure 2, after stimulation for 4 h, S100A8 and S100A9 increased the DNA-binding activity of NF-κB in PBMCs compared with that in untreated cells. As expected, LPS, which was used as a positive control, also increased this activity. These results were confirmed using an NF-κB p50 TransFactor ELISA kit (Clontech Laboratories), which allows direct measurement of the p50 subunit levels in the nucleus (**Fig. 2B**). NF-κB translocates into the nucleus after degradation of its inhibitor IκB-α, which occurs upon phosphorylation of the p50 subunit by the IKK complex [31]. Phosphorylation of the IKK complex and degradation of IκB-α in S100A8- and S100A9-stimulated cells was therefore investigated. Both proteins induced phosphorylation of IKKα/β/γ and degradation of IκB-α in PBMCs (**Fig. 2C** **)**, which was comparable to the effect induced by LPS. These results were consistent with the translocation of NF-κB induced by S100A8 and S100A9 in PBMCs.

### S100A8- and S100A9-mediated IL-6 and IL-8 Secretion Partially Depends on NF-κB Activation

**Figure 3:**
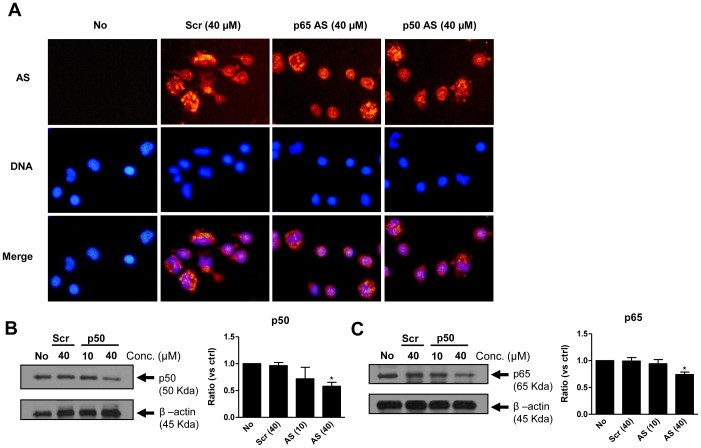
Inhibition of p50 and p65 using an antisense (AS) strategy in human PBMCs. Cells were treated for 4 h with p50 AS (10 or 40 µM), p65 AS (10 or 40 µM), the scrambled sequence AS (Scr 40 µM), or the equivalent volume of diluent (No). Cells were then fixed on coverslips for fluorescence microscopy (A) or lysed for immunoblotting of p50 (B) and p65 (C). Results are from one experiment out of 4 total experiments are shown. *p≤0.05, Dunnett’s multiple comparison test.

**Figure 4:**
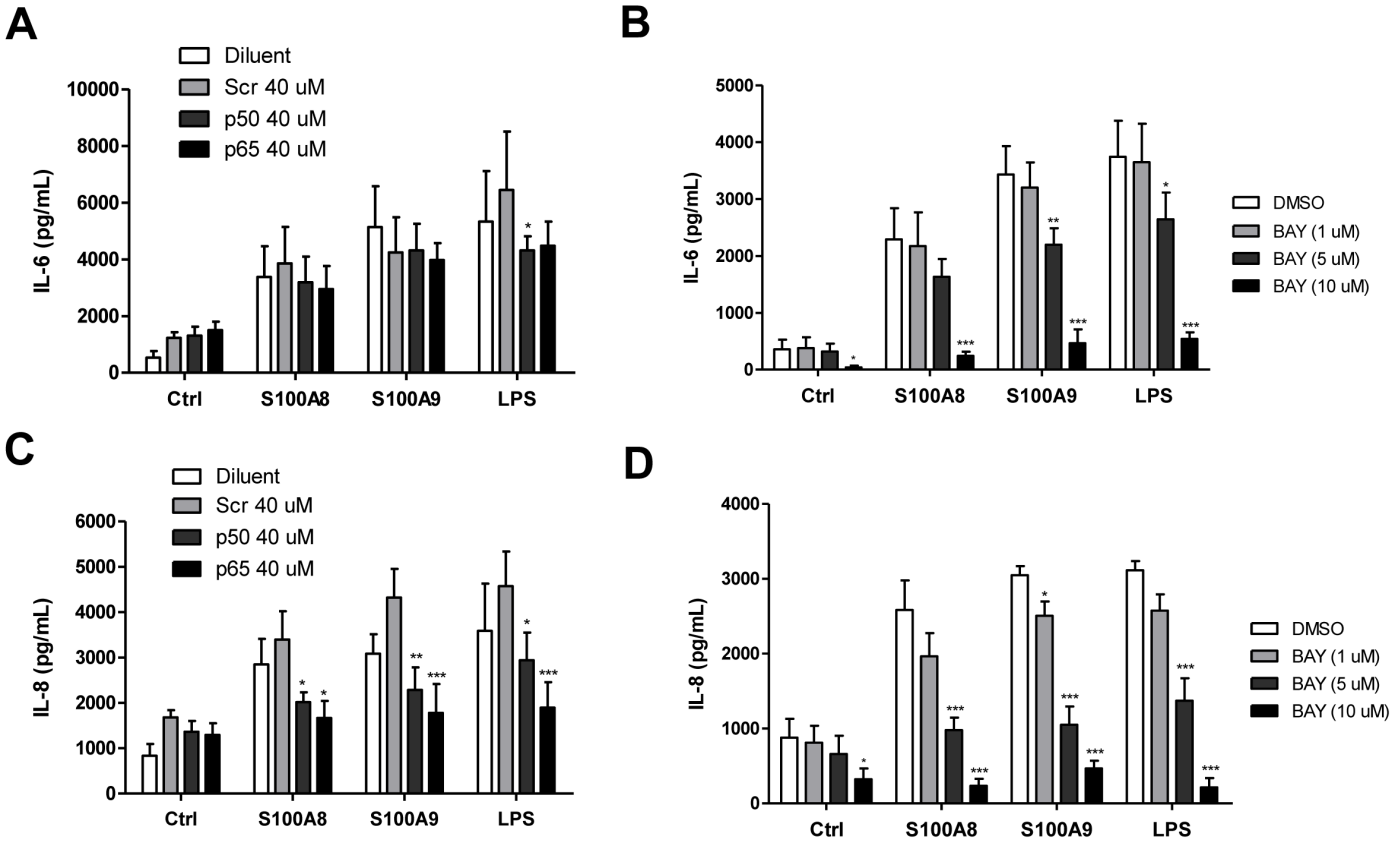
S100A8 and S100A9 induce IL-6 and IL-8 secretion via activation of NF-κB in human PBMCs. Secretion of IL-6 (A and B) and IL-8 (C and D) was evaluated using ELISA. Cells were pre-treated with 1–10 µM of BAY-117082 (or the equivalent amount of diluent) for 30 min or with antisense (Scr, p50, p65) for 4 h, followed by incubation with the indicated agonist for a total of 24 h. Supernatants were harvested and used for ELISA. Data are the mean ± SEM of at least four experiments performed on cells from different donors. *p≤0.05, **p≤0.01, ***p≤0.005, Dunnett’s multiple comparison test.

Next, we examined the role of NF-κB activation in S100A8- and S100A9-induced cytokine secretion. First, an antisense strategy was used to down-regulate p50 or p65 (Rel A) expression. As shown in Figure 3, p50 and p65 expression remained similar to that in control cells when scrambled antisense was added to the culture media. However, p50 antisense markedly decreased the expression of p50 (∼30%), whereas p65 antisense inhibited p65 less potently (∼15%). These results confirm that the p50 and p65 oligonucleotides were specific. Moreover, all antisense oligonucleotides were able to enter the cells at the same efficiency, as assessed by fluorescence microscopy. Decreased p50 and p65 expression resulted in reduced IL-8 secretion, confirming the importance of NF-κB for the activity of S100A8 and S100A9 (**Fig. 4**). Interestingly, inhibition of p50 and p65 did not significantly diminish IL-6 production. The IκB-α specific inhibitor BAY-117082 was also used to confirm these results. Addition of BAY-117082 almost completely inhibited IL-8 secretion, but IL-6 production was less affected.

### Translocation of NF-κB in Response to S100A8 and S100A9 Depends on ROS Production

**Figure 5:**
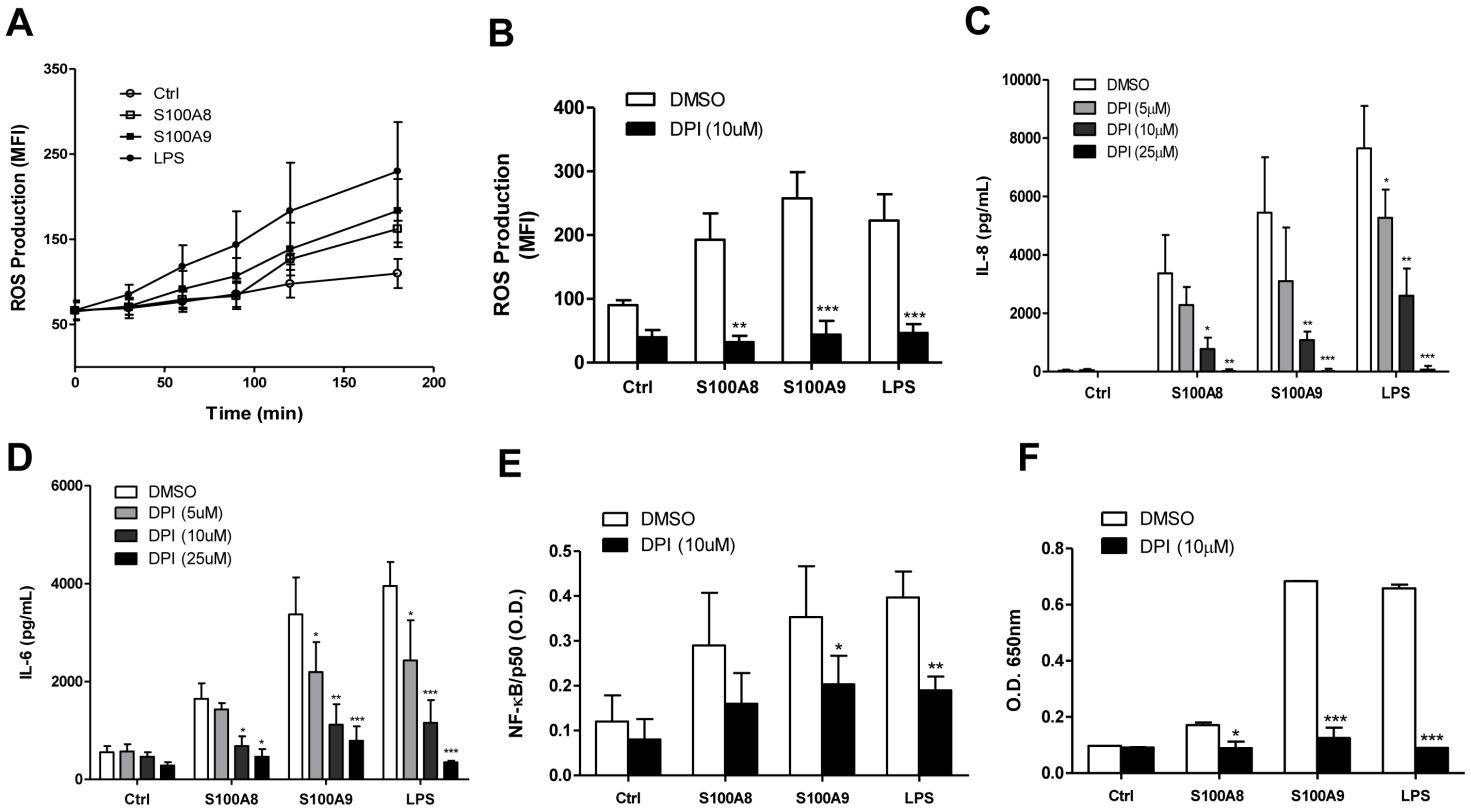
NF-κB translocation and IL-8 production in S100A8- or S100A9-treated cells are dependent on ROS production. Cells were stimulated for different time periods and ROS production was assessed by oxidation of H_2_DCFDA (A and B). In some experiments, cells were pre-treated with DPI for 30 min (B-F). ROS production was measured after 180 min (B). IL-6 and IL-8 secretion was assessed using ELISA (C and D). Translocation of NF-κB was assessed using the TransFactor p50 kit (E). NF-κB activity was followed with THP1-XBlue cells (F). Data are the mean ± SEM of three experiments performed on cells from four different donors (A-E). Data are the mean ± SEM of three independent experiments (F). *p≤0.05, **p≤0.01, ***p≤0.005, Dunnett’s multiple comparison test.

ROS actively participate as the second messenger in the induction of several genes in numerous physiological and pathological conditions [reviewed in [32]–[34]], and function, at least in part, through the activation of NF-κB [35], [36]. Because S100A8 and S100A9 trigger ROS production in other myeloid cells, including human neutrophils [11], the role of ROS in S100A8 and S100A9-induced NF-κB activation was investigated. First, the production of ROS in monocytes was measured using the dichlorodihydrofluorescein diacetate (H_2_DCFDA) probe. S100A8 and S100A9 induced a slow accumulation of ROS over time (**Fig. 5A**), with ROS production becoming detectable after 90 min of stimulation with S100A9 and after 120 min of stimulation with S100A8. Next, we used the flavoprotein inhibitor DPI, which inhibits ROS generation (**Fig. 5B**) from NADPH oxidases and mitochondria, and monitored the production of IL-8 and IL-6, as well as the activation of NF-κB. DPI was found to block the production of IL-8 (**Fig. 5C**) and IL-6 (**Fig 5D**) in a dose-dependent manner. Translocation of NF-κB was evaluated by measuring the p50 subunit levels in the nucleus in the presence of 10 µM DPI. DPI significantly reduced the p50 subunit levels in the nucleus of cells stimulated with S100A9 or LPS (**Fig. 5E**). A similar trend was observed when cells were exposed to S100A8. To confirm the negative role of DPI in S100A8- and S100A9-mediated NF-κB activation, the THP1-XBlue™ cell line reporting NF-κB activity was used. As expected, DPI strongly inhibited the activation of NF-κB in S100A8-, S100A9-, and LPS-treated cells (**Fig. 5F**). Again, S100A9 was found to be a more potent activator than S100A8.

### S100A8 and S100A9 Regulate the NLRP3 Inflammasome

**Figure 6:**
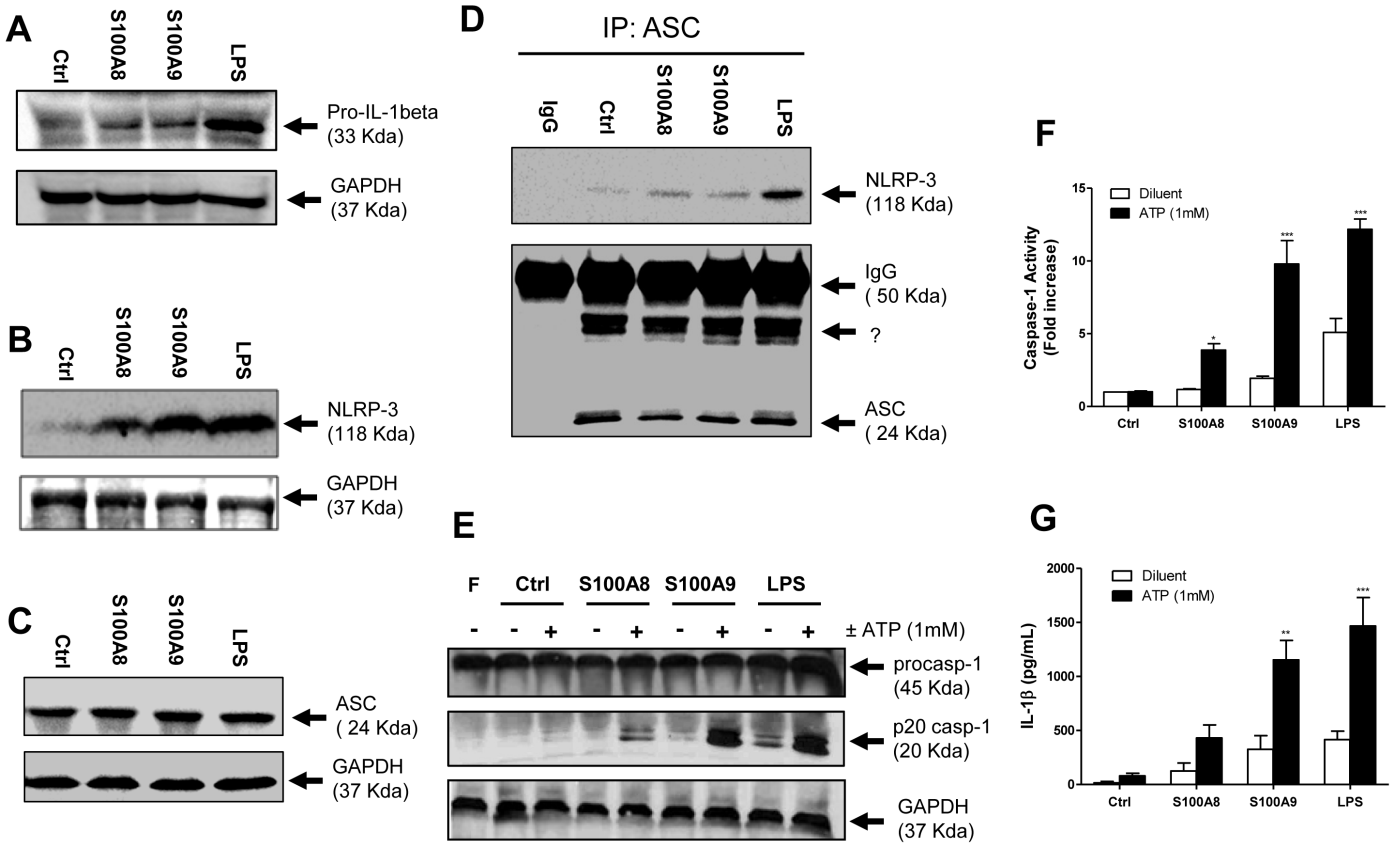
S100A8 and S100A9 regulate the NLRP3 inflammasome. Cells were incubated for 4 h with S100A8 (10 µg/ml), S100A9 (10 µg/ml), LPS (1 µg/ml), or the equivalent volume of buffer (Ctrl). For some experiments, ATP (1 mM) was added for 30 min (D–G). Cells were harvested and lysed with Laemmli’s buffer for immunoblotting of pro-IL-1β (A), NLRP3 (B), ASC (C) or caspase-1 (E). Cells were lysed with caspase-1 lysis buffer for the caspase assay (F). ASC immunoprecipitates were loaded on SDS-PAGE for NLRP-3 and ASC western blot analysis (D). Supernatants were harvested before performing IL-1β ELISA (G). Results are from one experiment that is representative of at least three others (A–E). Data are the mean ± SEM of three experiments performed on cells from different donors (F–G). ?, non-specific band (as indicated in the manufacturer’s datasheet). *p≤0.05, **p≤0.01, ***p≤0.005, Dunnett’s multiple comparison test.

Caspase-1 processing is the ultimate step in the activation of the NLRP3 inflammasome and leads to the secretion of IL-1β, one of the cytokines up-regulated by S100A8 and S100A9 in human PBMCs (**Fig. 1**). The NLRP3 inflammasome is tightly controlled by a priming step that is dependent on NF-κB [37]. Because S100A8 and S100A9 induced ROS production that resulted in the activation of NF-κB, the roles of these proteins in NLRP3 inflammasome regulation and activation were investigated. The expression of pro-IL-1β and NLRP3 was first analyzed by western blot after 4-h stimulation with S100A8, S100A9, or LPS. Both S100A8 and S100A9 increased pro-IL-1β levels, albeit more modestly than LPS (**Fig. 6A**). However, NLRP3 expression was greatly increased following stimulation with S100A8 and S100A9 to levels comparable to those achieved by LPS stimulation (**Fig. 6B**). Pretreatment of cells with S100A8 or S100A9 followed by stimulation with ATP, a known inflammasome activator, increased the association between ASC and NLRP-3 compared to control cells without affecting the expression of the adaptor protein ASC (**Fig. 6C and D** **).** Next, the processing of caspase-1 into the active 10-kDa and 20-kDa subunits was monitored in the presence or absence of a second signal mediated by ATP. Cell priming with S100A8 or S100A9 alone was not sufficient to induce a strong cleavage of caspase-1 (**Fig. 6E**). However, caspase-1 cleavage was synergistically enhanced by the addition of ATP. Caspase-1 assays were performed to further confirm the ability of S100A8 and S100A9 to modulate the enzymatic activity of caspase-1. As expected, the catalytic activity of caspase-1 was enhanced by ATP and potentiated by the presence of S100A8, and to a greater extent, by S100A9 (**Fig. 6F**). Next, the ability of S100A8 and S100A9 to induce IL-1β secretion in the presence or absence of ATP was examined using ELISA. Interestingly, the levels of extracellular IL-1β released by monocytes exposed to ATP+S100A9 were similar to those in cells exposed to ATP+LPS; however, the priming with S100A8 was again less potent (**Fig. 6G**).

### NLRP-3 and ASC are Essentials for IL-1β Secretion in S100A8 and S100A9-treated Cells

**Figure 7:**
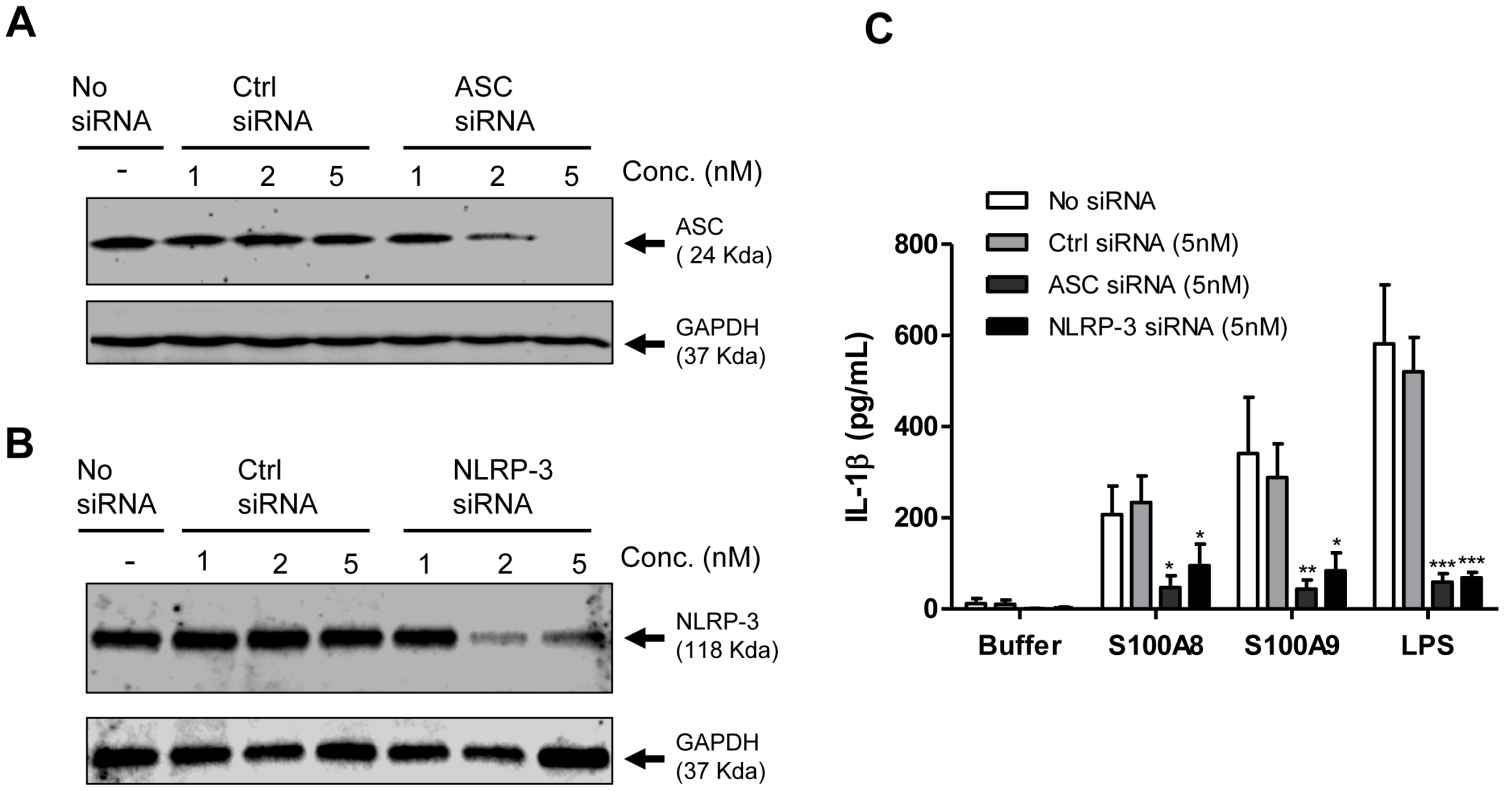
Role of ASC and NLRP-3 in the production of IL-1β by S100A8 and S100A9-treated human PBMCs. Cells were transfected with ASC siRNA, NLRP-3 siRNA, Ctrl siRNA or the equivalent volume of buffer (No) for 24 hours. Cells were then harvested and lyzed for western blot analysis (A and B) or stimulated for 4 hours with the indicated agonists and another 30 min with ATP 1mM. Supernatants were then harvested before performing ELISA (C). Results are from one experiment representative of three others (A and B). Data are the mean ± SEM of four experiments performed on cells from different donors (C). *p≤0.05, **p≤0.01, ***p≤0.005, Dunnett’s multiple comparison test.

Secretion of IL-1β is known to be regulated, at least in part, through the NLRP-3 inflammasome and its adaptor protein ASC [24], [25]. Therefore, the roles of NLRP-3 and ASC in the production of IL-1β in S100A8 and S100A9-primed cells were investigated. Addition of ASC-specific siRNA led to a 90% depletion of ASC (**Fig. 7A**) while siRNA against NLRP-3 reduced its expression by about 60% (**Fig. 7B**). Nevertheless, both silencing markedly reduced the secretion of IL-1β in S100A8 and S100A9-primed cells.

### ROS-mediated NF-κB Activation is Involved in NLRP3 Regulation by S100A8 and S100A9

**Figure 8:**
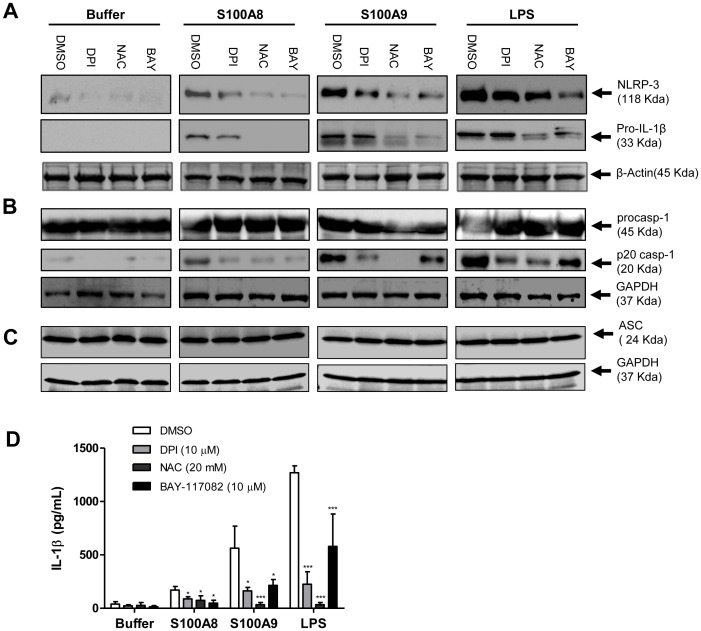
ROS- and NF-κB-mediated NLRP3 inflammasome priming by S100A8 and S100A9 in human PBMCs. Cells were pre-treated with various inhibitors (DPI, NAC, BAY-117082 or the equivalent volume of DMSO), then stimulated for 4 h with the indicated agonists. ATP (1 mM) was added for 30 min for caspase-1 immunoblots and for IL-1β ELISA (B and D). Cells were harvested and lysed in Laemmli’s buffer for immunoblotting of NLRP-3, pro-IL-1β, ASC and caspase-1.(A-C). Supernatants were harvested and IL-1β secretion was evaluated using ELISA (C). *p≤0.05, ***p≤0.005, Dunnett’s multiple comparison test.

NLRP3 activation is intimately linked to ROS generation [32]. Given that stimulation with S100A8 and S100A9 generated intracellular ROS in monocytes and promoted NF-κB activity, we investigated the possibility that oxidative stress is involved in S100A8- and S100A9-mediated NLRP3 inflammasome regulation and activation. DPI and the anti-oxidant NAC, a well-known ROS scavenger, were used to abrogate the effects of the oxidative stress induced by S100A8 and S100A9. DPI slightly reduced the expression of NLRP3 and pro-IL-1β in S100A8, S100A9-, and LPS-primed cells. However, NAC efficiently inhibited the expression of both NLRP3 and pro-IL-1β (**Fig.** **8A**). The inhibition was to a similar extent when cells were treated with the NF-κB inhibitor BAY-117082, suggesting that oxidative stress is important in the observed phenomenon (ASC expression levels remained constant, **Fig. 8C**). DPI and NAC strongly inhibited caspase-1 processing, whereas BAY-117082 had little impact on cells stimulated with S100A8, S100A9, or LPS in the presence of ATP (**Fig. 8B**). These results were corroborated by analyzing IL-1β release into the supernatant from the same samples. As illustrated in Figure 8D, NAC strongly impaired the secretion of IL-1β induced by S100A9 and LPS, and to a lesser extent, by S100A8. Again, NAC was a more potent inhibitor of IL-1β production than either DPI or BAY-117082.

## Discussion

In the last few years, studies have classified S100A8 and S100A9 as DAMPs. S100A8 and S100A9 were shown to be actively involved in the inflammatory response by regulating neutrophil functions [5]–[7], [10], [11], [38]. In this study, we further explored the roles of these two proteins by investigating cytokine production in human PBMCs. Both S100A8 and S100A9 induced the secretion of various pro-inflammatory cytokines in monocytes, including IL-8, IL-6, and IL-1β, through mechanisms dependent on ROS production and the activation of ROS-sensitive transcription factors, such as NF-κB. This study further demonstrated that S100A9, and to a lesser extent, S100A8, regulate the NLRP3 inflammasome by acting as priming agents. IL-1β secretion mediated by S100A8 or S100A9 alone was modest, but was greatly increased following caspase-1 activation via ATP.

The ability of S100A8 and S100A9 to promote expression of pro-inflammatory mediators including TNF-α, IL-1β, IL-6, and IL-8 through NF-κB activation has been previously reported in the cell line THP-1 and human macrophages [39], [40]. As in the present study, S100A9 more potently induced cytokine production compared with S100A8. This might reflect the use of different receptors, or a different affinity for their putative receptors TLR4 and/or RAGE [9], [41], [42]. However, the mechanisms by which these proteins increased NF-κB-mediated expression of pro-inflammatory cytokines remained unclear. Here, we report up-regulation of several other cytokines and chemokines, such as MIP-1α, MIP-1β, Gro-α, IL-23, G-CSF, and GM-CSF, in addition to TNF-α, IL-1β, IL-6, and IL-8. The expression of most of these cytokines depends on NF-κB activation, but other transcription factors could also be involved. This is highlighted by the fact that IL-6 secretion in response to S100A8 and S100A9 is less affected by NF-κB inhibition compared to IL-8 and IL-1β secretion, suggesting the involvement of other transcription factors in the secretion of IL-6. Indeed, multiple transcription factor-binding sites were identified in the human IL-6 promoter, including AP-1 [43]–[45]. AP-1 is intimately linked with the activation of mitogen-activated protein kinases (MAPKs) such as p38 and extracellular signal-regulated kinase (ERK1/2) [46]. We and others have previously demonstrated that S100A9 induced phosphorylation of p38, ERK1/2, Syk, and AKT in monocytes and neutrophils [10], [11], [40]. Interestingly, most of these kinases are regulated by redox-sensitive phosphatases such as tyrosine phosphatases (PTPs) and phosphatase and tensin homologs deleted on chromosome 10 (PTENs) [33], [47]. As previously observed in neutrophils, S100A9, and to a lesser extent, S100A8, induce ROS generation in monocytes that could potentially inactivate redox-sensitive phosphatases and thereby sustain cell activation.

ROS act as important second messengers and participate in numerous cellular functions through the regulation of redox-sensitive transcription factors, including NF-κB and AP-1 [33], [35], [48]. Our results show that the expression of IL-6 and IL-8 in monocytes primed with S100A8 or S100A9 depends largely on ROS generation, because DPI significantly impaired the expression of both cytokines. Similar effects were observed for IL-1β, the secretion of which is tightly regulated by caspase-1 activation. Signals triggering formation and activation of the inflammasome involve decreased intracellular K^+^ and the generation of oxidative stress [27], [49], [50]. These signals are typically provided by the engagement of toll-like receptors (TLRs) or the presence of endogenous stresses that modulate the ionic milieu and redox balance. Various inflammasomes have been identified in monocytes/macrophages such as the NLRP1, NLRC4, and NLRP3, which remains the most studied [51]. All of them converge toward caspase-1 activation and IL-1β secretion. The exact mechanisms responsible for the production and secretion of IL-1β remain unclear, but require two main steps. The first step involves the transcription and expression of proteins and/or precursors including pro-IL-1β and inflammasome subunits. The second step is inflammasome-mediated caspase-1 activation by stimuli such as ATP that generate robust K^+^ release [24], [28]. Tight control and regulation of the inflammasome is required to avoid excessive cytokine secretion. Therefore, regulation of the NLRP3 inflammasome occurs at the transcriptional and post-transcriptional levels. For instance, expression of NLRP3 requires a signal to be highly induced [52]. Interestingly, TLR signaling molecules are potent regulators of NLRP3 expression [37], [53]. In fact, efficient priming of cells by TLR signaling molecules is well documented, showing the importance of the priming step for efficient inflammasome activation [26], [54]. Our results demonstrate the importance of S100A8 and S100A9 in the regulation of the NLRP3 inflammasome, because these molecules upregulate NLRP3 and pro-IL-1β expression.

Various microbial-derived products can activate the NLRP3 inflammasome. Moreover, mitochondrial stresses, and host-derived molecules abundantly found in some metabolic and inflammatory diseases, such as excess ATP levels, oxidized low-density lipoproteins (LDLs), excess glucose, cholesterol crystals, monosodium uric acid (MSU), and high-mobility group box 1 (HMGB1) protein are also involved in NLRP3 inflammasome activation [55]–[58]. Here, we demonstrate that extracellular S100A8 and S100A9 are potent regulators of the NLRP3 inflammasome, and they act by influencing the redox balance in human monocytes. The mechanisms by which these proteins enhance ROS production are still unknown. However, considering that S100A8 and S100A9 partially mediate their effects through TLR4 activation [8], [40], we propose that intracellular signals induced by S100A8 and S100A9 are likely to be similar to LPS, which is known to trigger ROS production crucial for the expression of inflammatory cytokines through the activation of redox-sensitive transcription factors.

Release of mature IL-1β is greatly potentiated by priming of the NLRP3 inflammasome by NF-κB-driven protein synthesis stimulated by LPS [26]. However, long-term stimulation of monocytes in the absence of a co-signal, such as ATP, also leads to a slow activation of caspase-1 and liberation of IL-1β [59]–[61]. Our results show that S100A8 and S100A9 alone induce IL-1β secretion after long-term stimulation (24 h). This secretion was greatly enhanced by the addition of ATP. In fact, only 4 h of priming with S100A8 and S100A9 and 30 min of stimulation with ATP were sufficient to observe caspase-1 cleavage and IL-1β release. Uncontrolled activation of the inflammasome has been previously linked to several immune and non-immune diseases [58], [62]. For example, dextran sodium sulfate-mediated colitis and MSU-induced joint inflammation in a murine model of gout are less severe in mice deficient in caspase-1 or NLRP3 [63]–[65]. Similarly, mice deficient for the NLRP3 inflammasome adaptor protein, ASC, are protected from chronic obesity-induced pancreatic damage [66]. Interestingly, S100A8 and S100A9 concentrations have been correlated to disease activity in inflammatory bowel disease [15], [16], [67]–[69], gout [29], [70], obesity-mediated type 2 diabetes, and artheroslerosis [reviewed in [71]], suggesting a possible link between S100A8, S100A9, and exacerbated production of IL-1β.

Patients with hypercalprotectinemia have blood concentrations of S100A8/A9 that are 1000 to 10,000 times higher than the normal concentrations. These patients suffer from chronic systemic inflammation, presumably due to dysregulation of the S100A8/A9 metabolism [72]. This hereditary syndrome results from a mutation in the gene PSTPIP-1, which increases the binding of S100A8/A9 to PSTPIP-1. Interestingly, high concentrations of cytokines like IL-1β, IL-8, and TNFα were found in the serum of a patient with hypercalprotectinemia (unpublished observation). These cytokines are also detected in the supernatant of PBMCs stimulated with S100A8 and S100A9, suggesting that S100A8 and S100A9 could be responsible for dysregulation of cytokine production in hypercalprotectinemia patients. The higher concentrations of IL-1β detected in the serum of the hypercalprotectinemia patient also support the potential effects of S100A8 and S100A9 on NLRP3 inflammasome priming. This effect is likely not restricted to hypercalprotectinemia, because several cytokines actively involved in arthritis [73], including IL-6 and TNFα, were found to be upregulated by S100A8 and S100A9. In addition, blocking S100A9 resulted in diminished TNFα and IL-6 expression in a collagen-induced arthritis model [17].

In conclusion, the results presented in this study suggest a new role for S100A8 and S100A9 in redox-sensitive biological responses, including pro-inflammatory cytokine secretion. These findings also suggest a role of S100A8 and S100A9 in the pathogenesis of various inflammatory diseases.
